# Compound Heterozygote Friedreich Ataxia Patients With Covert Proximal *FXN* Gene Deletions

**DOI:** 10.1002/acn3.70408

**Published:** 2026-06-02

**Authors:** Michael P. Lazaropoulos, Morgan C. Devore, Christina Lam, Courtney Park, Sanjay Bidichandani, David R. Lynch

**Affiliations:** ^1^ Lewis Katz School of Medicine Temple University Philadelphia Pennsylvania USA; ^2^ Department of Pediatrics University of Oklahoma College of Medicine Oklahoma City Oklahoma USA; ^3^ Children's Hospital of Philadelphia Philadelphia Pennsylvania USA; ^4^ University of Pennsylvania School of Medicine Philadelphia Pennsylvania USA

**Keywords:** Friedreich ataxia, FXN gene, gene deletions, genetic testing, repeat expansions

## Abstract

We present Friedreich ataxia patients with frataxin gene deletions. Data and records were collected at the Children's Hospital of Philadelphia from patients enrolled in the FACOMS natural history study. Patients with proximal deletions initially diagnosed with only one GAA expanded allele had more severe disease than their homozygous expansion counterparts, including increased frequency of cardiomyopathy, diabetes, and optic neuropathy. Their phenotypes were like those of individuals with distal deletions and null pathogenic variants in the frataxin gene. Covert proximal frataxin gene deletions should be suspected when genetic testing fails to demonstrate two distinct expanded alleles in patients with severe phenotypes.

## 1 Introduction

Friedreich ataxia (FRDA) is an autosomal recessive disease leading to progressive cerebellar ataxia, sensory deficits, cardiomyopathy, scoliosis, and increased risk of diabetes [1]. It is caused by pathogenic variants in the *FXN* gene, which decrease production of functional frataxin protein [2]. The overwhelming majority (96%) of disease‐causing alleles involve expansion of a GAA triplet repeat in the first intron of *FXN*, which substantially decreases gene transcription [3, 4]. About 4% of disease‐causing alleles include other intragenic pathogenic variants in the *FXN* gene, such as missense variants and truncating variants (splice variants, nonsense variants, and insertion‐deletions of varying size).

Patients with non‐repeat expansion mutations are almost always compound heterozygotes, with one GAA repeat expansion allele [5]. Those with *FXN* missense variants vary clinically [6, 7, 8], while patients with large gene deletions (generally detected by Multiplex Ligation‐dependent Probe Amplification) often present with more severe features. The clinical course of all such patients is less predictable compared to that of patients homozygous for pure GAA expansion alleles.

A recent study identified novel disease‐causing alleles from those individuals appearing to carry two expanded alleles of the same size [9]. In roughly 50% of individuals with identical alleles by long‐range PCR, one of the two alleles is not amplified, leading to the false conclusion of two identical expanded alleles. Most of these individuals carry expanded composite repeats, but a few carry deletions of the proximal portion of the *FXN* gene. Here, we present the clinical features of FRDA patients who are compound heterozygotes for such novel deletions and one expanded GAA repeat allele and note their phenotypic similarities and differences to patients carrying traditional deletions and other null pathogenic variants that produce no frataxin.

## 2 Methods

All work was approved by the Children's Hospital of Philadelphia (CHOP) IRB under IRB 2609. Records from the Friedreich Ataxia Clinical Outcome Measures Study (FACOMS) at CHOP were reviewed for each of 4 patients identified as having a proximal deletion along with records of individuals followed at CHOP with various null pathogenic variants, including large distal *FXN* deletions. These data were compared to summary data from FACOMS subjects at CHOP carrying two expanded GAA alleles. Standard detection of expanded GAA repeats in the *FXN* gene was performed by Athena Diagnostics using polymerase chain reaction (PCR)‐based methods. Covert proximal *FXN* deletions were identified by long‐read whole genome sequencing (Oxford Nanopore Technologies), and confirmed by a custom PCR assay, as described [9]. Data analysis was performed using STATA v18 or Graphpad v10.6.1.

## 3 Results

### 3.1 Prototypic Patient With a Proximal Deletion

Patient 1 was diagnosed with FRDA at age six, presenting with ataxia in addition to his previous diagnosis of hypertrophic cardiomyopathy. He progressed to loss of ambulation at age 12 years, developed diabetes at 13, and severe vision loss at 16. Initial genetic testing determined he had inherited two GAA repeat expansion alleles of similar length. Years later, the patient's sister (Patient 2) developed FRDA at age 10 and was found to have inherited two pure GAA repeat expansion alleles of distinct sizes (490 and 990 repeats) from each of the carrier parents. Subsequent investigation of this discrepancy between the genetic analysis of these siblings revealed that Patient 1 had inherited only one GAA‐expanded allele while the remaining allele had undergone a *de novo* deletion of the proximal *FXN* gene encompassing the promoter and exon 1 [9]. Furthermore, Patient 2 had a much milder course of FRDA than her sibling Patient 1, with Patient 2 not developing cardiomyopathy or loss of ambulation at the time of the last clinic visit.

### 3.2 Other Patients With Null Variants

We then compared the clinical features of Patient 1 with those of individuals with similar deletions and with a variety of other *FXN* null variants, including large gene deletions (Table 1). All patients described in this report feature ataxia and at least mild dysarthria early in their clinical course. In general, patients with the proximal *FXN* deletion had early‐onset disease and early development of cardiomyopathy and scoliosis. Such features were also found in individuals carrying distal deletions and those carrying other null variants. Similarly, all patients with the deletions described developed poor weight gain and appetite, a documented endocrine feature of FRDA [10], with Patient 6 (c11_12delTC; Table 1) necessitating gastric tube placement for nutrition. Within these groups, though, selected individuals appeared relatively less affected (Patients 11, 14, 15; Table 1). All these individuals carried shorter GAA alleles.

**TABLE 1:** Clinical features of patients with various deletions of the *FXN* gene.

Identity	Allele 1: deletion	Allele 2: GAA allele length (triplets)	AOO	CMP (Age)	Diabetes (Age)	Chronic heart failure (age)	Wheelchair dependent (age)	Scoliosis surgery (age)	Vision loss (age)	Deceased (age)
Patient 1	Promoter, Exon 1, partial Intron 1	1020	6	6	13	NAP	12	12	16	N/A
Patient 2^a^	490	990	10	NAP	NAP	NAP	NAP	NAP	NAP	N/A
Patient 3	Promoter, Exon 1, partial Intron 1	920	8	9	NAP	16	14	15	NAP	16
Patient 4	Promoter, Exon 1, partial Intron 1	870	6	10	NAP	NAP	NAP	NAP	NAP	N/A
Patient 5	Promoter, Exon 1, partial Intron 1	890	1	4	NAP	NAP	14	15	NAP	N/A
Patient 6	c11_12delTC (frameshift)	635	4	11	NAP	NAP	11	NAP	NAP	N/A
Patient 7	c11_12delTC (frameshift)	Mosaic: 730 and 970	4	7	7	11	10	NAP	NAP	11
Patient 8	c11_12delTC (frameshift)	930	3	4	NAP	NAP	13	NAP	NAP	N/A
Patient 9	c.1A>C (start codon mutation)	1101	8	26	NAP	NAP	15	NAP	26	34
Patient 10	c.2delT (start codon mutation)	960	7	8	16	NAP	10	15	NAP	N/A
Patient 11	c.2delT (start codon mutation)	68	41	NAP	NAP	NAP	NAP	NAP	NAP	N/A
Patient 12	Exon 2, 3	600	10	11	NAP	NAP	14	NAP	NAP	31
Patient 13	Exon 5	663	7	17	NAP	NAP	NAP	13	NAP	N/A
Patient 14	Exon 5	483	10	15	NAP	NAP	NAP	NAP	NAP	N/A
Patient 15	Exon 5	533	9	15	NAP	NAP	NAP	NAP	NAP	N/A
Patient 16	Exon 5	1000	4	6	N/A	N/A	11	14	18	N/A
Patient 17	Exon 5	1000	3	5	N/A	14^b^	9	14	22	23

*Note:* Patients with various *FXN* deletions organized from the most proximal *FXN* deletions to the most distal.

Abbreviations: AOO, Age of Onset; CMP, Cardiomyopathy; NAP, not at present; N/A, not applicable.

^a^ Patient 2 is a patient without any deletions mutations and has two GAA‐expanded alleles to the *FXN* gene, included for the sake of comparison to sibling Patient 1.

^b^ Onset of heart failure following spinal fusion surgery.

Finally, we compared the clinical features of FRDA patients with null variants with those homozygous for two expanded GAA alleles (Table 2). Those with null variants had a lower age of onset, higher frequency of cardiomyopathy, diabetes, scoliosis repair, optic neuropathy, and age of death, even though the mean GAA1 (the shorter of the two GAA expanded alleles in homozygous patients) was similar (667 ± 235; *n* = 496) vs. 734 ± 228; *p* = 0.26, suggesting that the null allele leads to the relatively more severe phenotype. Such values were significantly more severe for the frequency of cardiomyopathy and for age at death and followed trends for the other values.

**TABLE 2:** Comparison of *FXN* ^*GAA/GAA*^ patients and all deletion patients (proximal, distal, null point mutations).

Identity	Allele 1	Allele 2	AOO	CMP (%)	Diabetes (%)	Scoliosis surgery (%)	Vision loss (%)	Deceased (%)
*FXN* ^*GAA/GAA*^, *n* = 478	666 ± 234	917 ± 280	12 ± 9 (*p* = 0.096)	61 (*p* = 0.0043)	8.0 (*p* = 0.14)	28 (*p* = 0.10)	10 (*p* = 0.073)	9 (*p* = 0.014)
Deletions, *n* = 16	Null	734 + 228	8.3 ± 8.6	93 (15/16)	19 (3/16)	43 (7/16)	25 (4/16)	32 (5/16)

*Note:* Continuous values were compared between *FXN* ^*GAA/GAA*^ patients and deletion patients by *t*‐test; percentages were compared by Fishers's exact test.

## 4 Discussion

The newly identified patients with proximal *FXN* gene deletions develop severe phenotypic features found in other individuals with non‐GAA‐repeat null variants. Initial genetic workup of these patients discovered only one expanded *FXN* allele, and thus, they were presumed to have two repeats of indistinguishable length. Thus, patients carrying two identical expanded alleles by commercial testing but with an unexpectedly severe (but otherwise typical) FRDA phenotype need further evaluation for an undetected deletion, just as single‐expanded‐allele patients with a normal GAA allele and a severe FRDA phenotype should prompt a search for a null, non‐GAA variant. In contrast, a single expanded allele associated with a normal GAA allele and an atypical phenotype directs one to a missense variant, though exceptions to this pattern occur.

The present findings stress that, while conventional approaches treat GAA1 as the primary determinant of severity, in extreme cases (null variants), GAA2 (the longer of two GAA expansion alleles in homozygous patients) can further refine FRDA severity. Most statistical models of disease severity do not include GAA2 sizes, as GAA1 length tends to provide the best index of residual *FXN* transcript (as seen in Patient 11). Depending on the calculation method, the use of GAA1 alone generates *R* ^2^ values of 0.4–0.5 for the prediction of different parameters. Though GAA2 is difficult to account for in many models, as its values are linked to GAA1 values, GAA2 length could explain some of the remaining variance in FRDA symptomatology.

## Author Contributions

M.P.L. and D.R.L. in conceptualization and writing. C.P. in data acquisition. M.C.D., C.L., and S.B. provided data and detailed information on the genetics of Patients 1 through 5.

## Funding

This work was supported by a FARA Graduate Research Fellowship (M C D), by FARA Grants to S.B. and D R L, and the US Department of Defense grants (USAMRAA) HT94252510541 (S.B.) and HT94252510542 (D R L).

## Ethics Statement

This study was approved by the Children's Hospital of Philadelphia IRB‐ Protocol number 2609.

## Consent

All subjects provided informed consent in advance of any procedures being performed.

## Conflicts of Interest

The authors declare no conflicts of interest.

## Data Availability

Data will be made available upon reasonable request.
